# Suppressed phase transition and giant ionic conductivity in La_2_Mo_2_O_9_ nanowires

**DOI:** 10.1038/ncomms9354

**Published:** 2015-09-18

**Authors:** Wei Liu, Wei Pan, Jian Luo, Andy Godfrey, Gang Ou, Hui Wu, Wei Zhang

**Affiliations:** 1 State Key Lab of New Ceramics and Fine Processing, School of Materials Science and Engineering, Tsinghua University, Beijing 100084, China; 2 Department of NanoEngineering, Program of Materials Science and Engineering, University of California, San Diego, La Jolla, California 92093-0448, USA

## Abstract

Improving the ionic conductivity of solid electrolytes at low temperatures represents a major challenge and an opportunity for enabling a variety of solid-state ionic devices for energy conversion and storage, as well as for environmental protection. Here we report a giant ionic conductivity of 0.20 Scm^−1^, achieved at 500 °C, in the La_2_Mo_2_O_9_ nanowires with a bamboo-wire morphology, corresponding to a 1000-fold enhancement in conductivity over conventional bulk material. Stabilization of the high-temperature phase is observed to account for about a 10-fold increase in the conductivity. We further demonstrate that fast surface conduction in ∼3 nm thick, partially ordered, surface ‘amorphous' films, under strain on the curved surfaces of the nanowires (as a non-autonomous surface phase or complexion), contributes to an enhancement of the conductivity by another two orders of magnitude. Exemplified here by the study of the La_2_Mo_2_O_9_ nanowires, new possibilities for improvement of conductivity and for miniaturization of solid-state ionic devices by the careful use of one-dimensional nanomaterials can be envisioned.

## Abstract (web summary)

Solid oxide-ion conductors are crucial components in many energy and environment technologies. Here, the authors produce La_2_Mo_2_O_9_ nanowires with high ionic conductivity which is attributed to both the stabilization of the high-temperature phase and excess conduction in the disordered surface phases.

## Graphical abstract

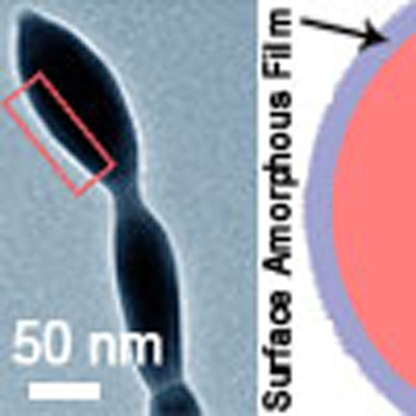

As a result of the continued drive towards increased energy efficiency and reduction of air pollution, solid oxygen-ion conductors have been developed as crucial components for various energy and environmental technologies, such as gas sensors, solid oxide fuel cells and oxygen-separation membranes. Interest in improving the ionic conductivity of solid oxygen-ion conductors, resulting in lower operating temperatures, has intensified over recent years, mainly driven by the necessity to replace conventional zirconia-based electrolytes with alternative materials with improved ion mobility at intermediate and low temperatures (below 750 °C)^1^,^2^. At the same time, rapid progress has been achieved in the development of miniaturized and portable electrochemical devices by the application of nano-structured materials. One-dimensional nanomaterials continue to be at the forefront of research into the application of functional micro-devices, both for the scientific community and for industry, on account of their exceptional performance^3^,^4^,^5^. As the size of a functional material is reduced to the nanoscale, the structure, phase stability and properties can all differ markedly from those in bulk counterparts, owing to both surface and nano effects^6^.

The use of La_2_Mo_2_O_9_ (LMO) as a solid oxygen-ion electrolyte was first reported by Lacorre *et al*.^7^ LMO exhibits fast oxygen-ion conducting properties at elevated temperatures, with an ionic conductivity slightly higher than stabilized zirconia^7^,^8^, the most widely used electrolyte at present. However, the ionic conductivity of LMO at low temperatures is poor, as a result of a reversible phase transition around 580 °C from the cubic β phase to a distorted monoclinic structure with a 2 × 3 × 4 supercell (the α phase), which causes a large reduction in ionic conductivity^7^,^9^,^10^. Moreover, LMO suffers from severe damage after cycling as a result of the volume change accompanying this first-order phase transition, thereby further limiting its practical application. It is highly desirable, therefore, to stabilize the high-temperature β phase at room temperature (RT). Various attempts have been made to achieve such a stabilization of the β phase by partial substitution of either the La site by cations such as Ca^2+^, Ba^2+^, Sr^2+^ (ref. 10), Nd^3+^, Gd^3+^ and Y^3+^ (ref. 11), or of the Mo site by Nb^5+^ (ref. 12) and W^6+^ (refs 9, 13). Although such doping provides some improvement in the low-temperature ionic conductivity, it does not yield significant improvement in the high-temperature ionic conductivity^13^,^14^.

Here we report for the first time stabilization of the high-temperature β phase down to RT, without use of dopants, in the LMO nanowires with a bamboo-wire morphology, prepared by electrospinning, an efficient, cost-effective and versatile method^15^. The nanowires show an enhancement in ionic conductivity over conventional LMO by three orders of magnitude, attributed both to the phase stabilization and to fast surface conduction. Moreover, the enhancement from surface conduction is greater than predicted from a simple composite rule, suggesting a curvature-dependent true size effect.

## Results

### Confirmation of the β phase in the nanowires at RT

**Figure 1:**
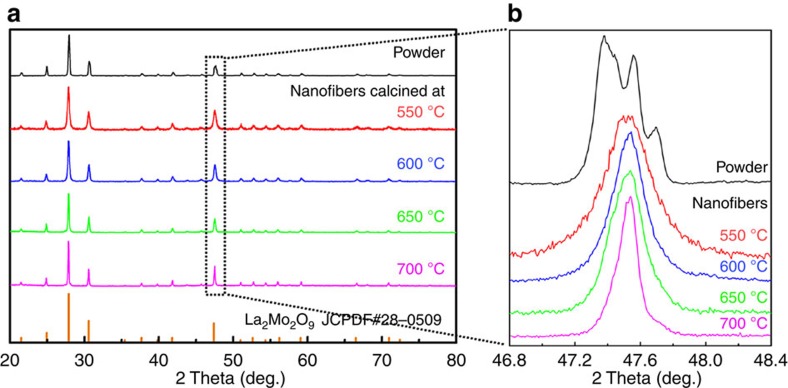
Phase identification of the LMO nanowires. (**a**) XRD patterns taken at RT of LMO nanowires calcined at various temperatures and of corresponding bulk LMO powder. (**b**) High-resolution XRD patterns showing the pseudo-cubic (321) reflection for the LMO powder whereas no peak splitting is seen for the nanowires.

As seen in Fig. 1a and Supplementary Fig. 2, powder X-ray diffraction (XRD) analysis suggests that both LMO nanowires calcined at temperatures between 550 and 700 °C, and the LMO powder, are crystallized and monophase. Step-scanning XRD (Fig. 1b) reveals a lattice constant for the LMO nanowires of 7.1565±0.0005 Å, which is close to the value of 7.155 Å in the PDF card. A more sensitive indicator for the presence of the monoclinic α phase is, however, a splitting of diffraction peaks in high-resolution XRD patterns^16^. As seen in Fig. 1b, we indeed find that high-resolution XRD patterns of the LMO powder show an obvious splitting of the pseudo-cubic reflection (321) at around 2*θ*=46.5–48.5°, indicating the presence of the monoclinic α phase. In contrast, no evidence of peak splitting is seen for the LMO nanowires, confirming the absence of the α phase and the complete stabilization of the β phase to RT in LMO nanowires.

**Figure 2:**
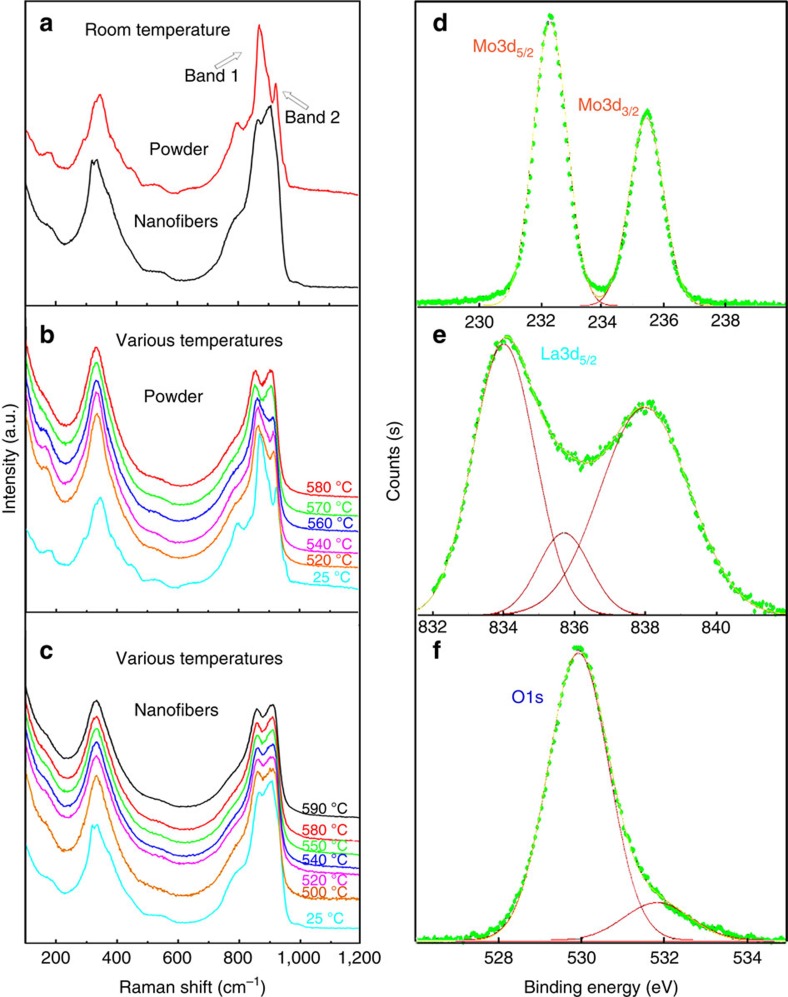
Raman and XPS spectra. Raman spectra for (**a**) LMO powder and LMO nanowires measured at RT; (**b**) LMO powder measured at various temperatures; and (**c**) LMO nanowires measured at various temperatures. Also show are XPS spectra for LMO nanowires calcined at 600 °C, showing the peaks of (**d**) Mo_3_d, (**e**) La_3_d and (**f**) O_1_s. See Supplementary fig. S12 for the complete spectrum.

Further insight into the difference between conventional LMO powder and the LMO nanowires is provided by Raman spectroscopy. Figure 2a–c shows Raman spectra for LMO powder and nanowires over a wide range of measuring temperature. The peak at around 866 cm^−1^ (Band 1) arises owing to the oscillation of oxygen vacancies, and the peak at 901 cm^−1^ (Band 2) is associated with oscillation of the Mo=O bond^17^. As shown in Fig. 2a, at RT the intensity of Band 1 is much higher than that of Band 2 for the LMO powder, whereas the opposite is true for the LMO nanowires, indicating a difference in phase structure^17^,^18^. Moreover, for the LMO powder a sudden decrease of the intensity of Band 1 (to approximately the same as that of Band 2) is observed at 570 °C (Fig. 2b), corresponding to the β-to-α phase transition. In contrast, Raman spectra for the LMO nanowires show no obvious variation over the temperature range from 25 to 590 °C (Fig. 2c), suggesting the absence of this phase transition.

Furthermore, X-ray photoelectron spectra (XPS) of the LMO nanowires (Fig. 2d) indicate benign stability, as a result of no reduction of Mo^6+^ takes place in the nanowires. Only two peaks at around 235.5 and 232.3 eV are seen in the spectra, corresponding to Mo3d_3/2_ and Mo3d_5/2_, respectively. According to the standard XPS database, these are both attributed to Mo^6+^, with no additional peaks corresponding to Mo^4+^ being seen^19^.

**Figure 3:**
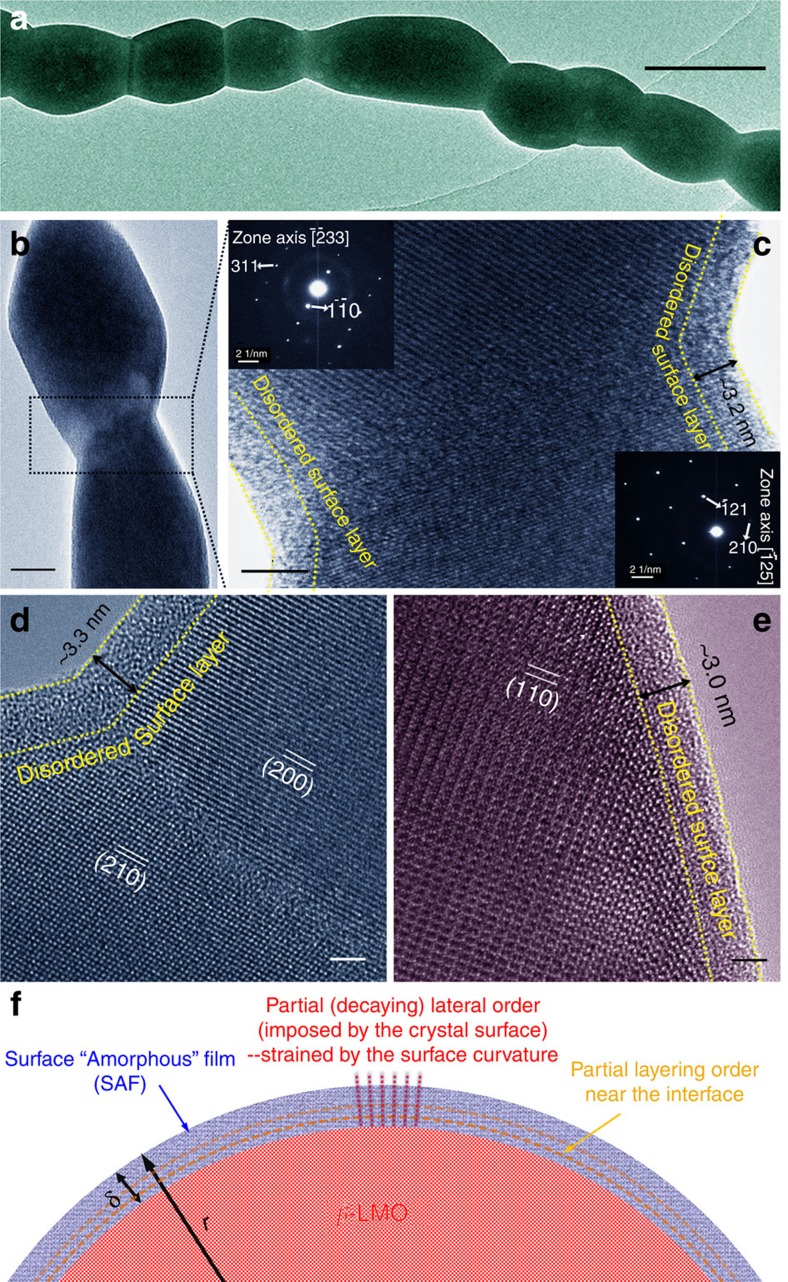
Structural characterization of the LMO nanowires. (**a**) Transmission electron microscopy image of an individual nanowire calcined 650 °C. (**b**) Transmission electron microscopy image of two grains. Also shown are high-resolution transmission electron microscopy images of nanowires calcined at (**c** and **d**) 600 °C and (**e**) 700 °C. The insets in panel (**c**) are the corresponding selected-area electron diffraction patterns. (**f**) Schematic illustration of the partial order in the glass-like structure near the crystalline grain in the SAF and the possible influence of surface curvature. It is well established that the crystal surface can impose both partial layering and lateral order to the glass structure at the crystal-glass interface, where the partial order decays away from the interface^28^,^35^,^36^. Recent modelling^37^ and experimental^38^ studies suggest enhanced ionic conduction along the crystal-glass interfaces (in the partially ordered region) that can be greater than that in either the crystal or glass phases. We hypothesize that such partial order in the SAF region, which is known to exist^28^,^35^,^36^, leads to enhanced ionic conduction. This is further enhanced by hoop strains arising from the high surface curvature, in agreement with our experimental observation of the curvature-dependent surface ionic conductivity.

As shown in Fig. 3a and Supplementary Fig. 4, the LMO nanowires have a bamboo-wire morphology consisting of single crystalline grains connected in series. Further analysis, documented in the Supplementary Information, reveals that LMO nanowires calcined at 600 °C or higher are pore-free and morphologically stable during subsequent conductivity measurements. Figure 3c–e show high-resolution transmission electron microscopy images, illustrating the excellent crystalline quality of the grains. The upper and lower insets are selected-area electron diffraction patterns taken along the 
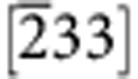
 and 
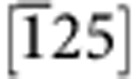
zone axes, respectively. The absence of any superstructure spot associated with the α phase again demonstrates that the LMO nanowires are fully formed of the cubic β phase^20^,^21^. Further confirmation of this is given by the absence of a thermal peak in differential scanning calorimetry curves (Supplementary Fig. 6) for the LMO nanowires, indicating that no phase transition takes place during heating. Collectively, the results from XRD, Raman spectroscopy, selected-area electron diffraction and differential scanning calorimetry all unanimously point to the fact that the β phase remains stable at RT.

We now consider explanations for the suppression of the β-to-α phase transition in LMO nanowires. In bamboo-like LMO nanowires, the β-to-α phase transformation can be thermodynamically suppressed if:


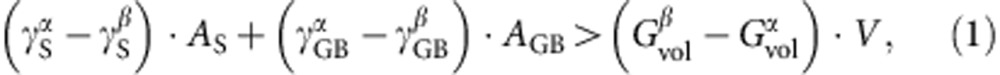


where for each phase *G*_vol_ is the volumetric free energy; *γ*_S_ and *γ*_GB_ are the surface (S) and grain boundary (GB) energies; and *V* and *A*_S_/*A*_GB_ are the volume and the surface and grain boundary areas, respectively. Note that in equation (1), small changes in the volume and the S/GB areas on phase transformation are neglected. Because stabilization of the β phase does not occur in nanoparticles of LMO with comparable size, the surface stabilization mechanism (whereby a positive value of the surface energy change, 
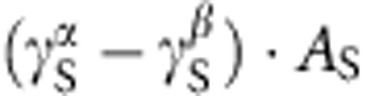
, offsets the negative volumetric energy change, 
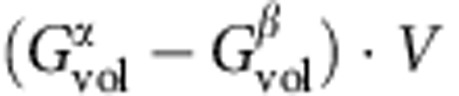
), which is now well established for Al_2_O_3_, TiO_2_, BaTiO_3_ and ZrO_2_ nanoparticles^22^,^23^, cannot be the sole reason for the observed stabilization of the β phase in the LMO nanowires. A positive value of the grain boundary energy contribution, 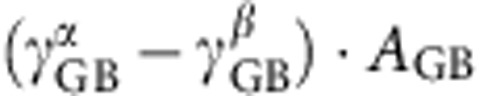, may, however, be a more effective and dominant driving force for stabilization of the β phase. In fact, it is reasonable to expect the α phase to have higher value of *γ*_GB,_ because the low symmetry of this phase, combined with a larger unit cell and ordered oxygen vacancies, imply more structural mismatching at the boundaries. Moreover, this hypothesis is supported by prior work^22^ suggesting that the β-to-α transition temperature is lower in sintered LMO with a smaller grain size.

It should be noted also that a similar suppression of phase transformation, also resulting in high conductivity at low temperatures, has been observed for α-AgI confined in glass^24^,^25^. Moreover, a recent study demonstrated that polyvinyl pyrrolidone (PVP)-coated α-AgI nanoparticles can be stabilized to RT (to maintain the high ionic conductivity) and the authors attributed the suppression of the α- to β-/*γ*-phase transition not only to a surface energy effect but also to the presence of defects and associated charge imbalance at the PVP/AgI interface^26^. In the current case, the LMO nanowires were annealed at ⩾600 °C in air so that the polymer component decomposed; yet, as we will elaborate later, nanoscale, La-Mo-O oxide-based, amorphous films formed on the surfaces of LMO nanowires (Fig. 3c–e), which may also induce defects and charge separation at/near the crystal-amorphous interfaces to contribute to the suppression of the β-to-α transition in LMO.

### Electrical property of the nanowires

**Figure 4:**
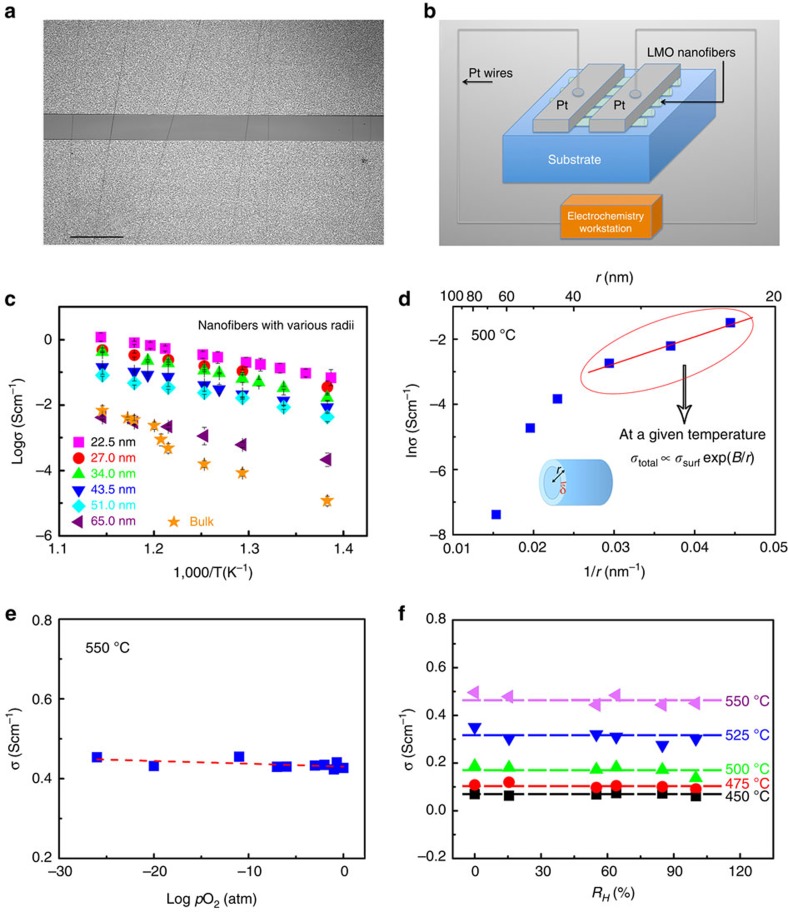
Measurement and analysis of the conductivities for the LMO nanowires. (**a**) Image of aligned wires placed on electrodes. (**b**) Schematic illustration of the configuration for the AC impedance spectroscopic measurements. (**c**) Arrhenius plots of the conductivities of LMO nanowires with various radii (made primarily by changing the salt-to-polymer ratio in the precursor solutions), together with the measured conductivities for corresponding bulk LMO. (**d**) Plot of ln(*σ*) versus 1/*r* for the nanowires (see Supplementary fig. S15 for the complete fitting with a model). Evidence of oxygen-ion conduction in LMO nanowires with an average diameter of 45 nm. (**e**) Conductivity of LMO nanowires versus oxygen partial pressure at 550 °C. (**f**) Conductivity of LMO nanowires versus humidity at various measuring temperatures.

For investigations of conductivity, LMO nanowires with consistent alignment, and covering a large area up to the wafer scale, were fabricated (Fig. 4a). Numerable nanowires were prepared on a quartz substrate by precise control of the collection time during electrospinning. To allow impedance spectroscopy measurements to be made, parallel Pt electrodes separated by a ∼50-μm-gap were sputtered on top of the nanowires (Fig. 4a). A schematic illustration of the assembly for testing the oriented nanowires is illustrated in Fig. 4b. The conductivity was determined from measured values of resistance, obtained by fitting the impedance spectra for the LMO nanowires (Supplementary Fig. 7). The temperature dependence of the total conductivity for the LMO nanowires as a function of radius is shown in Fig. 4c, together with data for corresponding bulk LMO. To measure the radius-dependent conductivities shown in Fig. 4c, six sets of LMO nanowires with different average radii (*r*=22.5–65.0 nm) were made primarily by changing the radius of the as-spun fibres via tuning of the salt-to-polymer ratio in the precursor solution during electrospinning. In addition, high-temperature (650 or 700 °C) calcination was used in two cases to achieve small adjustments of the nanowire radius (see Supplementary Table S1 for details of the specific fabrication conditions of the six sets of nanowires, and the Supplementary text for a discussion of the effects of calcination). It is important to note that all six sets of samples were calcined at temperatures no lower than 600 °C for sufficiently long durations to ensure that the LMO nanowires were fully crystallized, pore-free and morphologically stable; that is, no appreciable changes in morphology (including the average radius) took place during subsequent impedance measurements conducted at temperature up to 600 °C (as directly verified in Supplementary Figs 8a-d). Further experiments also verified no significant changes in the measured conductivity after as many as five heating and measurement cycles, as shown in Supplementary Fig. 8e.

For bulk LMO, an abrupt decrease in the conductivity by about one order of magnitude is seen, resulting from the phase transformation from the high-conductivity β phase to the low-conductivity α phase. In contrast, no such loss of conductivity is observed for the LMO nanowires, implying a suppression of the α-phase transition. The conductivity at 500 °C was measured as 0.22 Scm^−1^ for LMO nanowires of radius (*r*) of 22.5 nm and 8.38 × 10^−5^ Scm^−1^ for bulk LMO; at 600 °C the corresponding values are 1.18 Scm^−1^ and 6.66 × 10^−3^ Scm^−1^, respectively. The conductivity of LMO nanowires (*r*=22.5 nm) is therefore more than 2,000 times higher than that of the bulk α-phase (500 °C) and 200 times higher than that of the bulk β-phase (600 °C). Careful measurements showed that the conductivity of the quartz substrate without LMO nanowires was negligibly small (lower than the detection limit of the instrument) in the temperature range of 400–600 °C. Thus, the measured conductivities are from the nanowires.

The nature of the conductivity in the LMO nanowires, and in particular whether or not it is ionic, is of significant importance with respect to their potential application. Figure 4e shows the dependence of conductivity on oxygen partial pressure. The results reveal a constant conductivity over a wide range of *p*O_2_ values, suggesting that the conduction originates mainly from oxygen ions, and not from electron migration. In addition, Hebb–Wagner DC polarization measurements also confirmed that the conduction is largely ionic (with ionic transfer numbers of ∼0.95; see Supplementary Table 2). Moreover, Fig. 4f shows that the conductivity is almost unchanged over a large range of humidity at various temperatures, which indicates that protonic surface conduction should be negligible. It is concluded therefore that the LMO nanowires are ionic conductors.

Typical AC impedance spectra for LMO nanofibers (Supplementary Fig. 7) measured at various temperatures show only one well-defined semicircle at high and intermediate frequencies that can be ascribed to the total resistance. Grain and grain boundary responses for oxides typically show capacitance values of around 10^−12^ F and 10^−9^ F in impedance measurements, respectively^27^. Supplementary Table 3 lists the corresponding capacitance values for LMO nanowires measured at various temperatures. In each case the capacitance of the nanowires is closer to values expected for grains, suggesting that the grains provide a larger contribution to the total resistance than the grain boundaries.

## Discussion

In addition to the effect of phase stabilization, the further increase of the ionic conductivity with decreasing radius of the LMO nanowires should be attributed to fast surface conduction associated with the nanoscale dimensions of the wires (Fig. 4d, Supplementary Figs 14 and 15). As shown in transmission electron microscopy images of various LMO nanowires (Fig. 3c,d; Supplementary Fig. 9), surface ‘amorphous' films (SAFs) of a nearly constant thickness (3.0±0.3 nm), akin to equilibrium-thickness SAFs found in other oxides^28^,^29^,^30^,^31^, were observed to form on the nanowire surfaces. These SAFs were found on all six sets of LMO nanowires investigated, with the thickness largely independent of the nanowire radius (see Supplementary Fig. 9). These nanoscale SAFs can be considered as a two-dimensional ‘non-autonomous' surface phase following the terminology used by Defay and Prigogine^32^, also referred to as a ‘complexion' phase in more recent literature^33^ (see Supplementary text for an elaboration of the differing terminologies and for further discussion of SAFs). In contrast, similar intergranular films are absent at the grain boundaries (Fig. 3d). It can be noted that LMO powder synthesized by a sol-gel method and calcined at 600 °C also exhibits similar SAFs (Supplementary Fig. 10), but presumably disappear during sintering, as similar films are not seen at grain boundaries (see for examples Fig. 3d showing a grain boundary in a LMO nanowire). Thus, no improved conductivities due to these SAFs can be expected in sintered bulk polycrystalline specimens. Several recent studies have highlighted the possibility of using similar nanoscale non-autonomous interfacial phases (complexions) to achieve superior properties unattainable by bulk phases (for example, enhanced rate capability of Li-ion batteries^29^,^30^,^31^ and enhanced proton conductivity^34^), attributed at least in part to the presence of structures that are neither fully amorphous (despite being called SAFs) nor completely crystalline^28^,^35^,^36^. It has been suggested that in such materials the crystal surface can impose partial order into the ‘amorphous' structure of the nanoscale SAFs (Fig. 3f)^28^,^35^,^36^. More specifically, recent theoretical^37^ and experimental^38^ studies suggest that ionic conduction can be enhanced along the crystal-glass interfaces in such partially ordered regions, resulting in a value greater than those of both the individual bulk crystal and glass phases. Thus, we hypothesize that the observed increased ionic conductivity in the LMO nanowires is owing to enhanced ionic transport in the nanoscale SAFs, particularly in the partially ordered region near the crystal-glass interfaces (Fig. 3f).

Moreover, as shown in Supplementary Fig. 12, XPS revealed no impurities other than La, Mo and O in the SAFs. From an analysis of the XPS data, we measured the surface composition of LMO nanowires to be La_2.00_Mo_2.09_O_8.50_ (cf. the stoichiometric composition of LMO), indicating oxygen deficiencies on the nanowire surfaces. Electron energy-loss spectra (EELS) spectra of the LMO nanowires were recorded at the O-*K* edge and La-*M*_4,5_ edge, as shown in Supplementary Fig. 13a. From the EELS analysis of the nanowires (see Supplementary text for details) it is reasonable to hypothesize that oxygen vacancies or vacancy-like complexes form in the partially ordered region in the glass-like SAFs near the crystalline grains, leading to enhanced ionic conduction.

It is important to recognize that many possible mechanisms can lead to enhanced ionic conductivities, such as space-charge, strain and other effects^26^,^39^,^40^,^41^,^42^,^43^,^44^,^45^. To some extent, the current case is analogous to the study of PVP-coated α-AgI, where both change of the interfacial energies and possible presence of charged defects at the crystal-amorphous interfaces may help stabilizing the high-temperature, high-conducting phase^26^. A most recent study showed that carrier–carrier coulombic interactions can have an important role in the ionic conduction in complex oxides^45^. In the current case, we further observed curvature-dependent excess conductivities (above the conductivities of the bulk high-temperature β-LMO), which were not observed for PVP-coated AgI and other cases and could not be explained by prior models. The origin of the observed curvature-dependent excess conductivities can be best explained by the fast conduction in SAFs strained on curved surfaces (Fig. 3f), supported by the high-resolution transmission electron microscopy observation of SAFs and measured conductivities as a function of the radius of the LMO nanowires, which will be discussed below and elaborated in the Supplementary Information.

A more careful examination of the conductivity of the nanowires as a function of the surface-to-volume ratio shows that the enhanced ionic conductivity is not a ‘trivial size effect' as defined by Maier^40^,^41^,^42^,^43^ (see Supplementary Figs 14-15 and the Supplementary text). Maier *et al*.^40^,^41^,^42^,^43^ cited an ‘accelerating' increase of conductivity as the dimension approaches the Debye length, owing to enhanced carrier concentration in the space change region, as a case of a ‘true size effect'. For the LMO nanowires, the effect of wire radius on conductivity can be understood following the idea that the diffusion coefficient of atoms at a curved surface increases with the surface curvature^46^. Such a curvature effect can also manifest in the partially ordered region of the SAFs that are strained on the curved surfaces of the nanowires, leading to an exponential increase of the surface conduction with curvature, as shown in Fig. 4d and Supplementary Fig. 14. A detailed derivation of a phenomenological model to account for this effect is given in the Supplementary text, from which an equation for conductivity (Supplementary Equation 10 in the Supplementary Text) can be derived that fits the experimental data well (Supplementary Fig. 15). Although the exact form for variation of conductivity with radius is likely to be more complex, the agreement with our model (Supplementary Fig. 15) suggests that a curvature effect can nevertheless explain the observed nonlinear increase of conductivity with surface area.

In summary, we have prepared LMO nanowires with a bamboo-wire microstructure by electrospinning. We report, for the first time to our knowledge, stabilization of the β-phase at RT without the use of dopants. Moreover, we observed a nonlinear increase of conductivity with decreasing nanowire radius, which contributes a significant increase in ionic conduction. As a result of these two effects, the nanowires have a giant ionic conductivity at 500 °C of 0.22 Scm^−1^, about 2,000 times higher than that of conventional bulk LMO. This work opens the door for novel developments in the use of one-dimensional nanomaterials for a myriad of potential electrochemical applications, such as single-chamber micro-solid oxide fuel cells (Supplementary Fig. 16), gas separation, hydrogen production, oxygen sensors for automobiles, and other solid-state ionic devices operating at low temperatures.

## Methods

### Fabrication of LMO nanowires and assembly of specimens

(NH_4_)_6_Mo_7_O_24_·6H_2_O (>99%) and La(NO_3_)_3_·6H_2_O (>99.95%) of corresponding molar fractions were dissolved in water, followed by addition of citric acid to chelate the metal cations. PVP (molecular weight=1,300,000) was then added and a transparent solution was obtained by vigorous stirring in a water bath at 50 °C. To control the diameter of the LMO nanowires, we chose various of salts to PVP weight ratios in the precursor solution. To conduct electrospinning, the precursor solution was loaded into a 1 ml plastic capillary tube with a stainless-steel needle. A high voltage of 15 kV was applied by dipping a charged silver thread into the precursor solution. A copper bridge with a gap of 1 cm was used as the cathode; this was placed 15 cm beneath the needle tip, allowing the collection of uniaxially aligned electrospun PVP/(NH_4_)_6_Mo_7_O_24_/La(NO_3_)_3_ wires (Supplementary Fig. 1).

The as-spun wires were subsequently transferred to quartz substrates (1 × 1 cm) by lifting out the wires from underneath the copper bridge, after which they were calcined at temperatures of either 550, 600, 650 or 700 °C in air for 2 h. In addition, six sets of LMO nanowires, each with different average radii, were synthesized. All were calcined at temperatures no lower than 600 °C such that the nanowires were fully crystallized and pore-free and the morphology (including the average radius) was stable during all subsequent measurements. The specific parameters for fabrication of these six sets of LMO nanowires are listed in Supplementary Table 1.

To allow electrical conductivity measurements to be made, parallel Pt electrodes separated by a ∼50 μm gap were sputtered on top of nanowires placed on a quartz substrate (Fig. 4a). A schematic illustration of the assembly and device for testing of the oriented LMO nanowires is illustrated in Fig. 4b. The collection time during electrospinning was controlled precisely to enable obtaining a sufficient (but not excessive) number of well-aligned and non-overlapping LMO nanowires on the substrate. The number of the nanowires between the electrodes is around 100, which varies according to different sample.

### Fabrication of LMO bulk specimens

For comparison with the nanowires, LMO powders were prepared by a sol-gel synthesis using starting materials of (NH_4_)_6_Mo_7_O_24_·6H_2_O, La(NO_3_)_3_·6H_2_O and citric acid. A transparent viscous gel was obtained by stirring these materials together at 90 °C. The dry gel was calcined in the temperature range of 550–900 °C for 3 h in air. The as-synthesized LMO powder was then ground, sieved, dry pressed into pellets, and sintered at 1,200 °C for 12 h in air. Pt was sputtered as an electrode for AC measurement of the bulk LMO.

### Materials characterization

XRD experiments were conducted using a D/max-2500 H diffractometer (Rigaku, Akishima-Shi, Japan; using Cu *K*_α_ radiation of 0.15406, nm) for phase identification, crystal structure analysis and determination of the lattice constants. Additional high-resolution XRD spectra were taken for the range of 2*θ*=46.5–48.5° at RT using a step-scanning mode. The phase structure and phase transition characteristics of the wires were probed by Raman spectroscopy (Model LabRAM HR800, HORIBA Jobin Yvon, Paris, France). The element valence state was determined by XPS using a PHI Quantera SXM system (ULVAC-PHI, Kanagawa, Japan). Specimens for transmission electron microscopy were examined using a JEM-2010 F microscope (JEOL, Tokyo, Japan). EELS spectra were obtained using a JEM-ARM200F (JEOL, Tokyo, Japan) aberration-corrected electron microscope, operated at 200 kV.

### Electrical measurements

Values of *p*O_2_ were measured and controlled using a ceramic oxygen sensor (assembled from a commercially sourced YSZ tube; measurements were conducted in a separated high-temperature chamber). Impedance spectra of the LMO nanowires were measured under conditions covering a range of humidity (using dry air bubbled through a H_2_O bath) and temperature. A digital humidity metre was used to measure the relative humidity. To further clarify the nature of the charge carriers in LMO nanowires, DC Hebb–Wagner polarization measurements were carried out. The results were used to determine the ionic transport number following refs 47, 48, 49. Specifically, DC Hebb–Wagner polarization measurements were performed using an ion-blocking electrode to determine the electronic conductivity of the LMO nanofibers. The blocking electrode was prepared from a low melting point glass such that it fully covered one of the Pt electrodes forming the measurement cell. To avoid damage of the LMO nanofibers, we selected a glass with a melting point of 450 °C and carefully printed the glass paste on the electrode. After calcination at 500 °C for a short time, we confirmed that the blocking electrode was fully covered by the glass by inspection using an optical microscope. A potentiostatic DC voltage ranging from 0.2 to 1.1 V was applied between the reversible and blocking electrode, and the obtained current–voltage dependence was used to calculate the electronic conductivity. From these data, the electronic and ionic transfer numbers were calculated and are listed in Supplementary Table S2. Electrical conductivity was investigated using AC impedance spectroscopy (IM6, Zahner, Kronach, Germany) over a frequency range from 0.1 Hz to 8 MHz in air.

The conductivity, *σ*, of the nanowires was calculated from the resistance (*R*) measured at various temperatures using the following relationship:


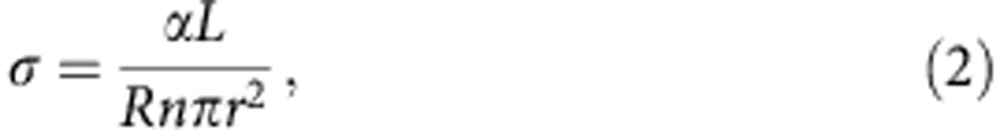


where *L* is the distance between the two Pt electrodes, *α* is a geometrical factor (that is equal to the mean of 1/sin*θ*, where *θ* is the measured angle between the nanowire and parallel electrodes), *n* is the number of LMO nanowires (typically about 100), and *r* is the average radius of the nanowires.

## Additional information

**How to cite this article:** Liu, W. *et al*. Suppressed phase transition and giant ionic conductivity in La_2_Mo_2_O_9_ nanowires. *Nat. Commun.* 6:8354 doi: 10.1038/ncomms9354 (2015).

## Supplementary Material

Supplementary Information Supplementary Figures 1-17, Supplementary Tables 1-5, Supplementary Discussion and Supplementary References
