# Supplementary material for: Suppressed phase transition and giant ionic conductivity in La2Mo2O9 nanowires
Source: Nat Commun. 2015 Sep 18;6:8354. doi: 10.1038/ncomms9354 (PMC4595754; doi:10.1038/ncomms9354)
Supplement: Supplementary Information — Supplementary Figures 1-17, Supplementary Tables 1-5, Supplementary Discussion and Supplementary References [file ncomms9354-s1.pdf]

Supplementary Figures

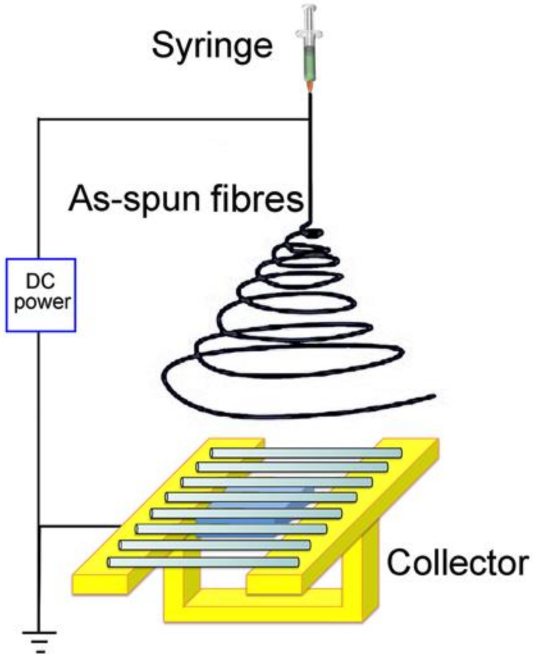

Supplementary Fig. 1. Schematic illustration of the electrospinning set-up.

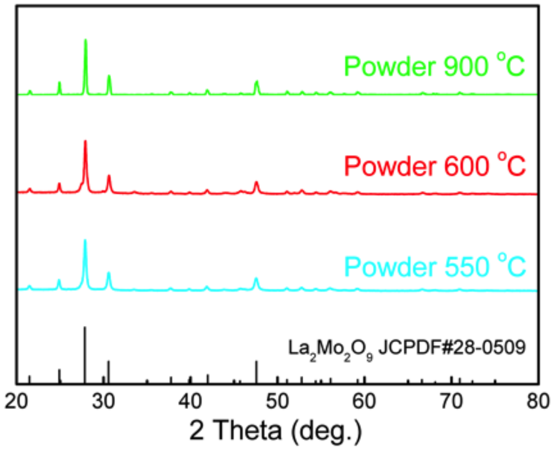

Supplementary Fig. 2. XRD patterns of LMO powders calcined at various temperatures.

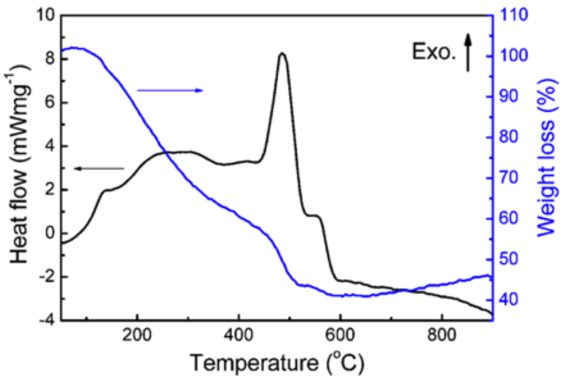

Supplementary Fig. 3. DSC/TG curves for the as-spun LMO fibers.

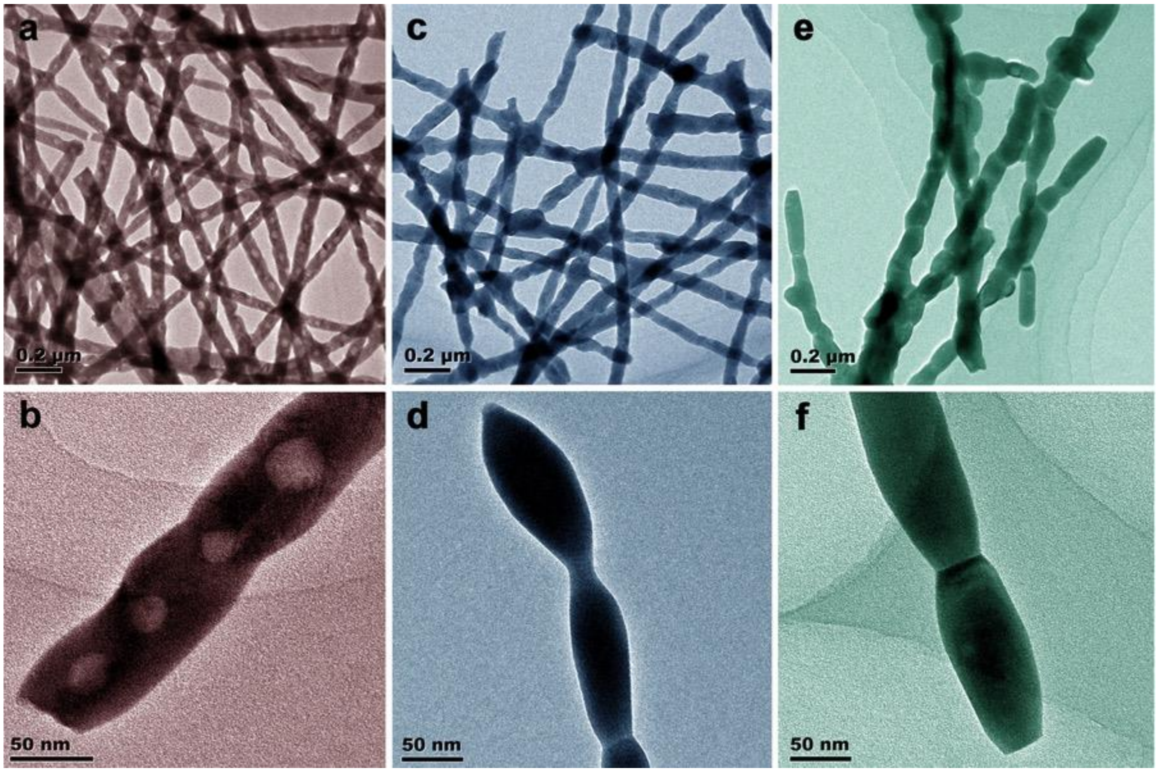

Supplementary Fig. 4. TEM images of LMO nanowires calcined at various temperatures: (a and b) 550 °C; (c and d) 600 °C; and (e and f) 650 °C. It is important to note that all LMO nanowires used for the subsequent conductivity measurements were calcined at temperatures no lower than 600 °C for sufficiently long durations to ensure that they were fully crystallized and pore-free, and to ensure that the diameter, morphology, and conductivities of these nanowires remained stable during subsequent measurements (as demonstrated in Fig. S8). Porous LMO nanowires (calcined at 550 °C) are shown in panel (a and b) for comparison only (i.e., no conductivity measurements were conducted on such porous nanowires).

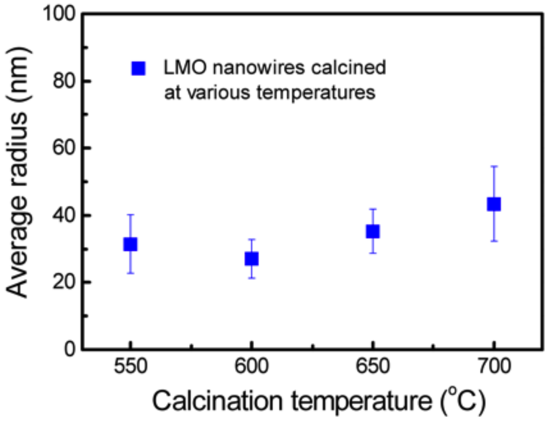

Supplementary Fig. 5. Average diameter of LMO nanowires calcined at various temperatures (made using a fixed salt-to-polymer weight ratio of 0.6 in the precursor solution). The decrease in radius from the calcination temperature of 550 °C to 600 °C results from removal of pores; slight coarsening is seen for annealing at 650 to 700 °C. It should be noted that different nanowire radii were achieved mainly by changing the salt-to-polymer ratio in the precursor solution during electrospinning of the nanowires, and high-temperature (≥ 650 °C) calcination was used only for small adjustments of the radius (Table SI), for which the morphological change was “locked in” (in part due to the strong pinning effects at the surface grooves in the bamboo-wire structure) during subsequent measurements at lower temperatures. Likewise, for the measurements of radius-dependent conductivity shown in Fig. 1a in the main text, all six sets of samples were calcined at temperatures no lower than 600 °C, to ensure no changes in radius or morphology took place during the subsequent measurements made at lower temperatures.

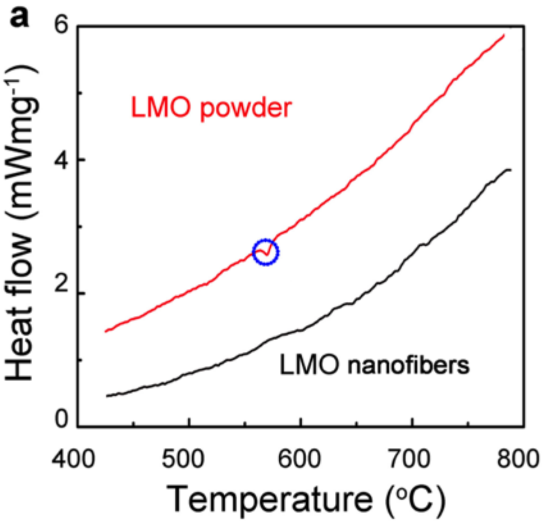

Supplementary Fig. 6. DSC curves of LMO nanowires with an average diameter of 45 nm (calcined at 600 °C before the DSC measurement) and of LMO powder made by the sol-gel method. The α-to-β phase transformation signature is evident for the LMO powder but not for the nanowires.

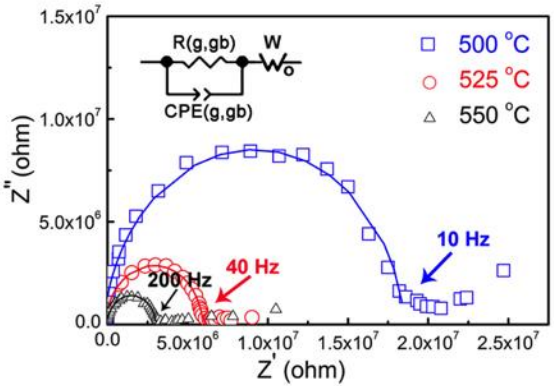

Supplementary Fig. 7. Experimental and fitted impedance spectra for LMO nanowires with an average diameter of 45 nm, measured at different temperatures. The inset (top-left corner) shows the equivalent circuit used to fit the spectra.

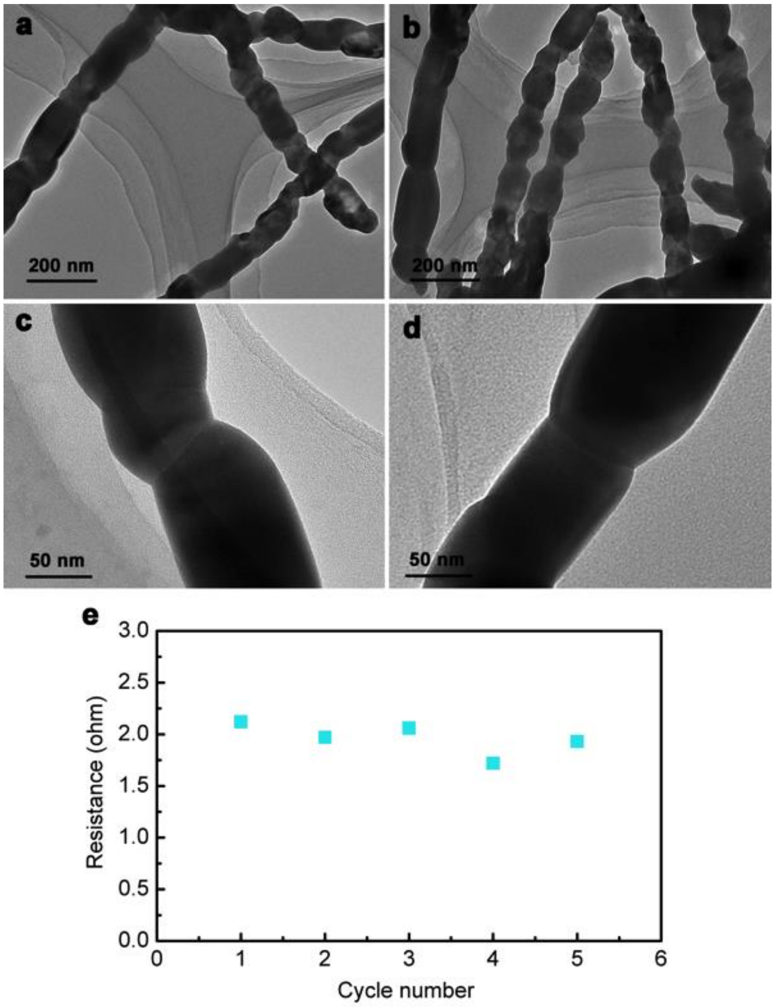

Supplementary Fig. 8. TEM images of the LMO nanowires (a and c) before and (b and d) after five cycles of heating and conductivity measurement. (e) The measured resistance of the LMO nanowires did not change significantly over five heating and measurement cycles, indicating the phase and morphology were stable during the conductivity measurements. The specimen was cycled between 400 and 600 °C and the resistance values plotted here were measured at 500 °C.

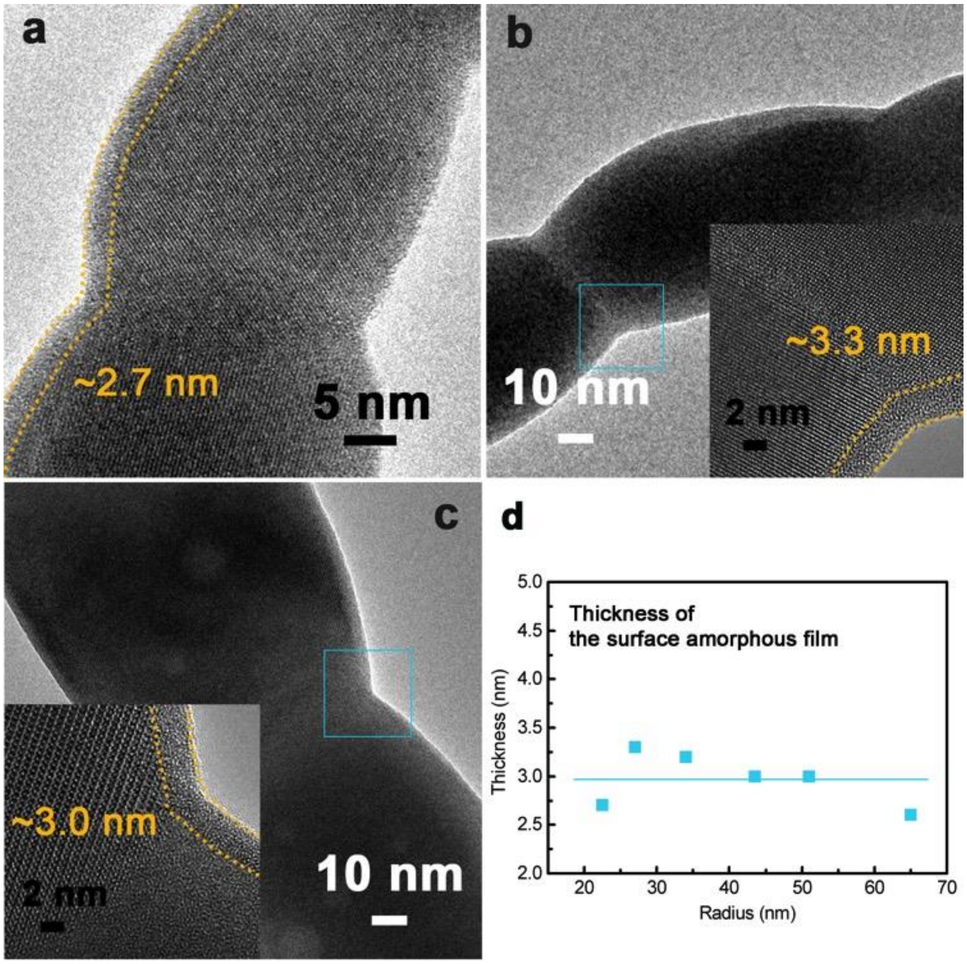

Supplementary Fig. 9. (a-c) High-resolution TEM (HRTEM) images of the LMO nanowires of various diameters, showing SAFs of almost constant thickness (in addition to the HRTEM images shown in Figs. 3c-3e in the main text). (d) Plot of measured thickness of the SAFs *vs.* radius for the nanowires.

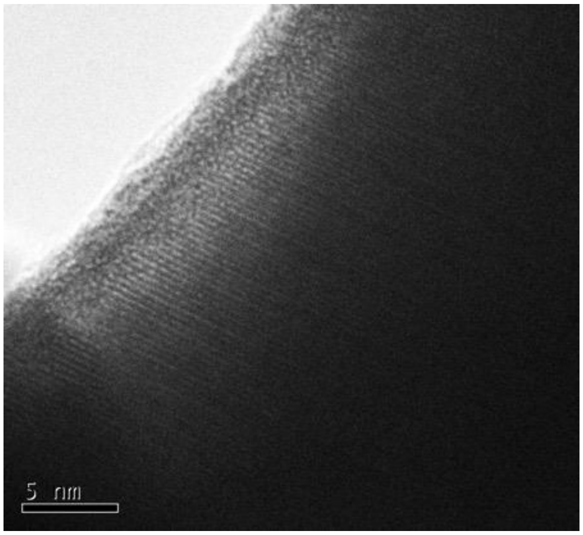

Supplementary Fig. 10. HRTEM image of the surface of a LMO powder particle calcined at 600 °C, showing a SAF similar to those seen in the LMO nanowires.

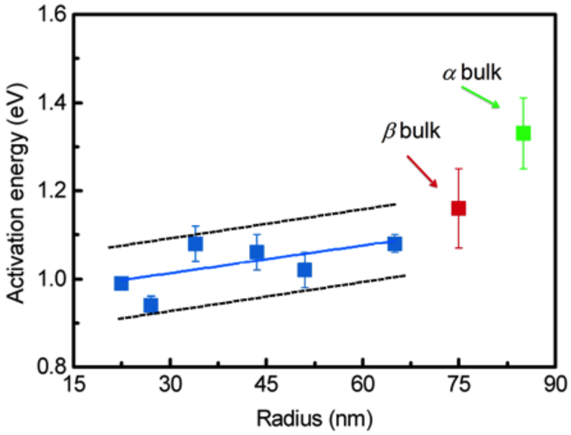

Supplementary Fig. 11. Activation energies for the LMO nanowires of various radii. Also plotted are values for the bulk β and α phases (measured from sintered dense polycrystalline material).

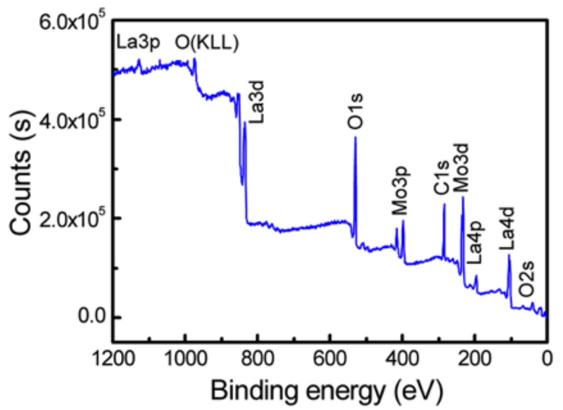

Supplementary Fig. 12. The complete XPS spectrum for the LMO nanowires with an average diameter of 45 nm.

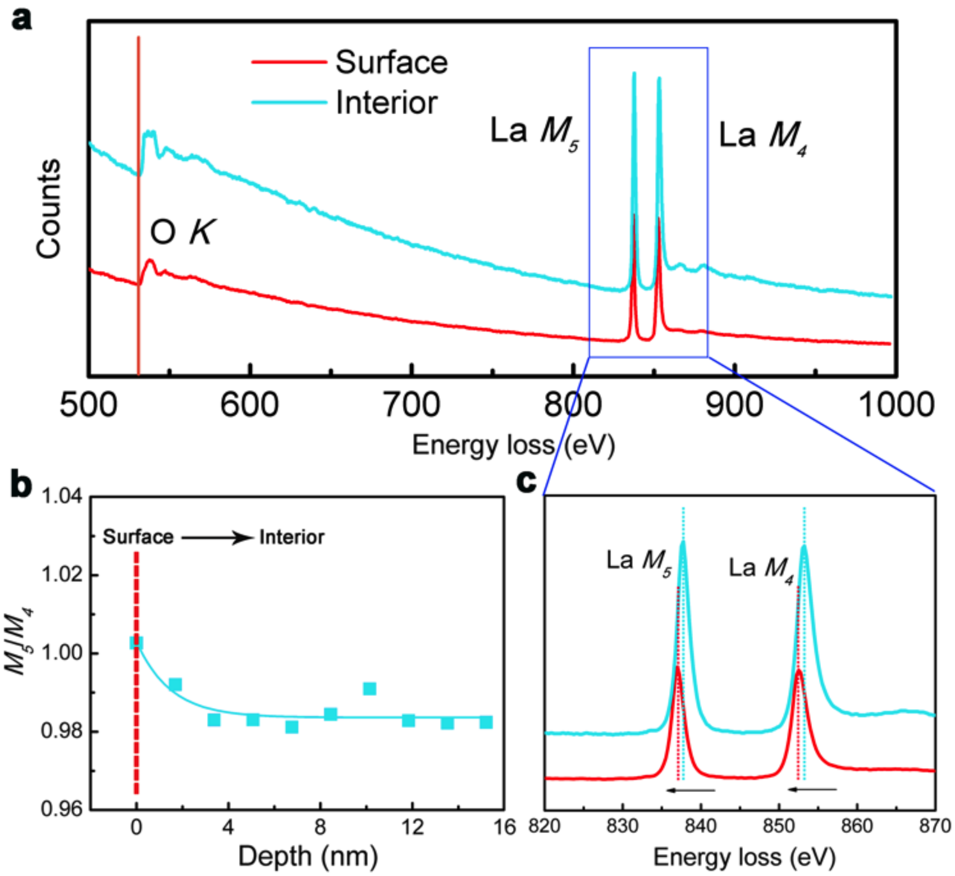

Supplementary Fig. 13. EELS analysis of the LMO nanowires. (a) EELS spectra from the surface and interior of a LMO nanowire calcined at 600 °C, showing the O-*K* and La-*M* edges. (b) Depth profile of M_5/M_4 for a LMO nanowire. (c) expanded view of the region from 820 eV to 870 eV of EELS spectra collected from the surface and interior of a LMO nanowire, showing a red shift from the interior to the surface.

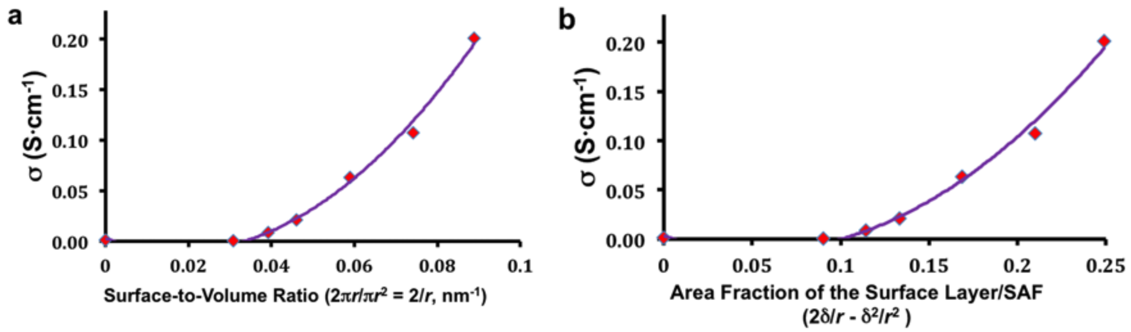

Supplementary Fig. 14. Variation of ionic conductivity at 500 °C with (a) surface-to-volume ratio and (b) area fraction of the SAF ($\delta = 3$ nm) for LMO nanowires. Noting that in this analysis, the geometrical factor α, which is roughly a constant that scales the conductivities uniformly (so it does not affect the analysis), is not considered.

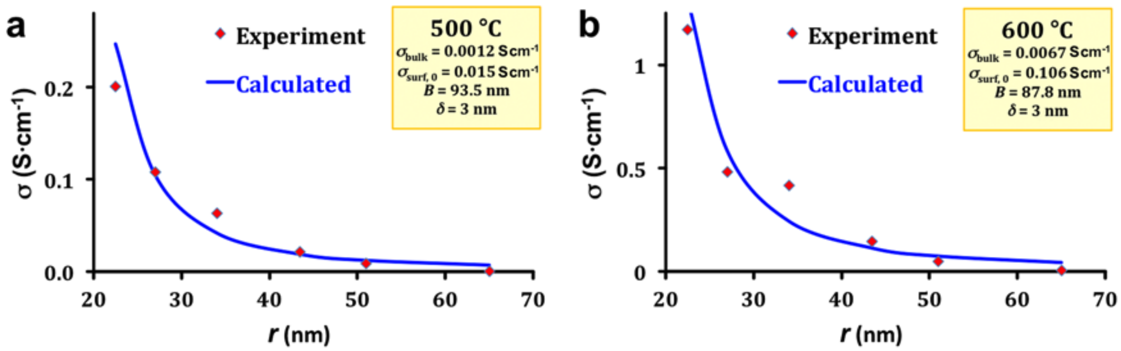

Supplementary Fig. 15. Measured ionic conductivity (σ) *vs.* radius (r) of LMO nanowires, together with calculated curves based on our model (Eq. (11)) for (a) 500 °C and (b) 600 °C. Noting that in this analysis, the geometrical factor α, which is roughly a constant that scales the conductivities uniformly (so it does not affect the analysis), is not considered.

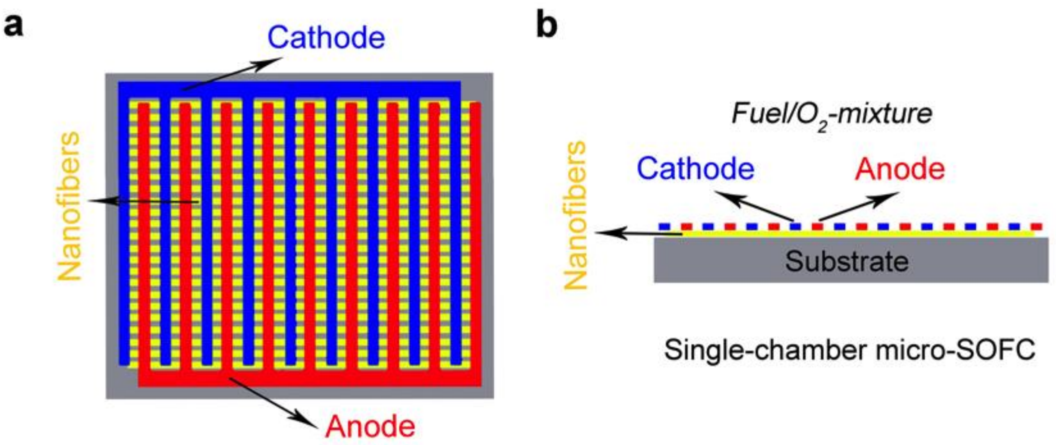

Supplementary Fig. 16. Schematic of a proposed application of the LMO nanowires as a single-chamber micro-SOFC using oxygen-ion conducting nanowires. (a) Top view. (b) Cross-sectional view.

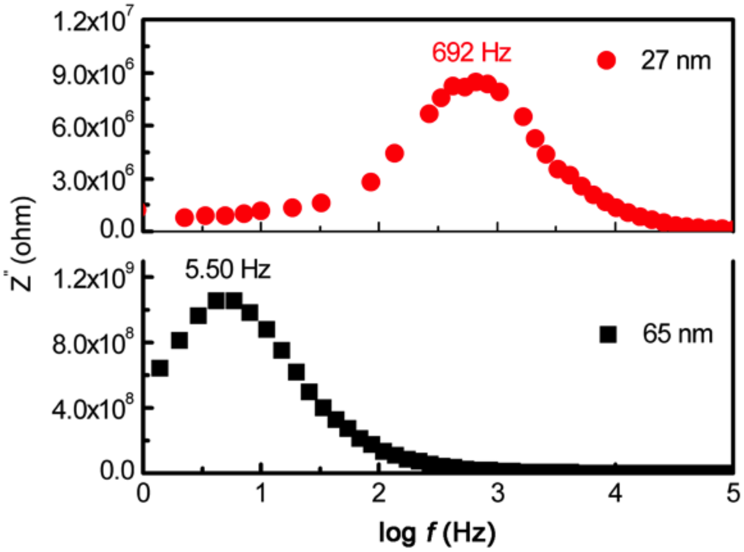

Supplementary Fig. 17. Plots of Z'' *vs.* $\log_{10}(f)$ for LMO nanowires with radii of 27 nm and 65 nm (measured at 500 °C). The two-order of magnitude increase in the position of the frequency maximum (f_{max}) for the 27-nm-radius nanowire compared to that for the 65-nm-radius nanowire further substantiates the measured increase in conductivity, as this method is less sensitive to errors due to geometrical factors.

Supplementary Tables

Radius (nm)	22.5	27	34	43.5	51	65
**Salt-to-polymer Ratio**	0.4	0.6	0. 6	0.6	0.8	1
**Calcination Temperature (°C)**	600	600	650	700	600	600

Supplementary Table 1. Experimental parameters for fabrication of the six sets of nanowires with different radii used in the conductivity measurements. It should be noted that different nanowire radii were achieved mainly by changing the salt-to-polymer ratio in the precursor solution during electrospinning of the nanowires. High-temperature (650 or 700 °C) calcination was additionally used to achieve a small adjustment of the radius in two cases, for which we believe that the morphological change was "locked in" (in part due to the strong pinning effects at the surface grooves in the bamboo-wire structure) during the subsequent measurements at lower temperatures. It is also important to note that all six sets of samples were calcined at temperatures no lower than 600 °C and therefore we expect no change in radius or morphology during the subsequent measurements at lower temperatures (as verified by the experimental data shown in Fig. S8).

Measurement Temperature (°C)	Electronic Conductivity (Scm^{-1})	Total Conductivity (Scm^{-1})	Electronic Transport Number	Ionic Transport Number
**450**	0.003	0.069	0.044	0.956
**500**	0.013	0.220	0.059	0.941
**550**	0.031	0.558	0.056	0.944

Supplementary Table 2. DC Hebb-Wagner polarization measurement results for LMO nanowires with an average diameter of 45 nm at various measuring temperatures.

$T_{measured}$ (°C)	Resistance (ohm)	CPE	n	Capacitance (F)
**500**	1.860×10^{7}	4.254×10^{-11}	0.9417	2.734×10^{-11}
**525**	6.032×10^{6}	2.185×10^{-11}	0.9324	1.143×10^{-11}
**550**	3.094×10^{6}	2.471×10^{-11}	0.9251	1.147×10^{-11}

Supplementary Table 3. Capacitance values for the LMO nanowires with an average diameter of 45 nm measured at various temperatures (T_{measured}).

Element	Average area ratio (Surface/Interior)
**La**	0.69
**O**	0.50

Supplementary Table 4. Average integrated-area ratios of the surface region to interior area calculated from the La *M*-edge and O *K*-edge for LMO nanowires with an average diameter of 45 nm.

T (°C)	*r* = 22.5 nm	*r* = 27 nm	*r* = 34 nm	*r* = 43.5 nm	*r* = 51 nm	*r* = 65 nm	bulk
**350**							3.11E-07
**400**							3.84E-06
**450**	6.85E-02	3.50E-02	1.68E-02	8.52E-03	4.32E-03	2.13E-04	1.17E-05
**462**	9.51E-02						
**475**	1.31E-01		3.28E-02	1.39E-02	8.69E-03		
**490**	1.73E-01		4.78E-02				
**500**	2.20E-01	1.09E-01	6.35E-02	2.13E-02	1.64E-02	6.13E-04	8.38E-05
**515**	2.81E-01		9.30E-02	3.07E-02			
**525**	3.41E-01	1.56E-01	1.12E-01	4.12E-02	2.36E-02	1.13E-03	1.56E-04
**550**	5.58E-01	2.37E-01	2.09E-01	7.21E-02	3.34E-02	2.13E-03	4.71E-04
**560**							2.33E-03
**565**	6.63E-01		2.57E-01	8.32E-02			
**575**	7.99E-01	3.34E-01		1.06E-01	4.73E-02	2.91E-03	3.52E-03
**580**							3.95E-03
**600**	1.18E+00	4.84E-01	4.18E-01	1.46E-01	8.04E-02	4.16E-03	6.66E-03

Supplementary Table 5. Measured conductivity (σ) *vs*. temperature (*T*) for bamboo-like LMO nanowires of different average radii (*r*), and for bulk LMO (unit: Scm^{-1}).

Supplementary Discussion

Supplementary Discussion 1. The Effects of Calcination

It can be seen from Fig. S4 that LMO nanowires calcined at temperatures of 550, 600 and 650 °C all show similar bamboo-wire structures. The LMO nanowires calcined at 550 °C contain some porosity (Figs. S4a and S4b). This is in agreement with the DSC/TG results, which indicate that the LMO nanowires calcined at 600 °C and above exhibit nearly complete crystallization and are pore-free (Fig. S3). All conductivity measurements were carried out on nanowires that were calcined at 600 °C or higher and hence on samples that were pore-free with good crystallinity and morphological stability.

The variation of LMO nanowire radius as a function of calcination temperature, made using a fixed polymer-to-precursor ratio, is shown in Fig. S5. The decrease in radius as the calcination temperature is raised from 550 °C to 600 °C results from the removal of pores. The radius is similar for calcination temperatures of 600 °C and above, with a slight coarsening for annealing at 650 to 700 °C. Thus, tuning of nanowire radius (to give six sets of samples with r = 22.5-65.0 nm) was mainly achieved via changing the polymer-to-precursor ratio, with high-temperature (650 or 700 °C) calcination used only for small adjustments of the radius (Table S1), such that the additional morphological change was “locked in” (in part due to the strong pinning effects at the surface grooves in the bamboo-wire structure; see Fig. S4) during subsequent measurements at lower temperatures. Moreover, all six sets of samples were calcined at 600 °C or higher for a sufficiently long duration to crystallize and densify LMO, and to ensure that no significant further coarsening (resulting in changes in the average diameter and/or the microstructure) took place during subsequent AC impedance measurements, which were conducted at temperatures up to 600 °C.

The morphological stability of the nanowires during heating and measurement was additionally verified experimentally by repeating the heating and measurement process up to five times while examining the morphology *ex situ* and measuring the conductivity *in situ*; see Fig. S8 and related discussion for further details.

Supplementary Discussion 2. Electron Energy Loss Spectroscopy (EELS) Analysis of LMO Nanowires

EELS was performed to detect differences in the chemical state of the surface and interior of the nanowires. In order to achieve the required high spatial resolution, aberration-corrected scanning transmission electron microscopy (AC STEM) was carried out at 200 kV in conjunction with EELS, using a JEM-ARM200F microscope (JEOL). The EELS spectra were collected under the spectroscopic imaging mode, where the microscope was configured to produce a nanoscale STEM probe at a chosen position on the sample. An EELS spectrum was acquired at each of a succession of probe positions as the beam was scanned across a survey region, and from this a 3D spectrum-image data cube was obtained pixel by pixel (1.6 nm/pixel in our case). Therefore, each EELS spectrum was correlated to its position in the real space. To enhance the signal-to-noise ratio, the EELS spectra shown in Figs. S13a were obtained by accumulating data from 114 pixels, corresponding to a 3 pixel (along radius direction) × 38 pixel (along wire growth direction) window, in selected regions of the interior and surface of the nanowires. A 1 pixel × 40 pixel window was used for collecting the white-line ratio profile shown in Fig. S13b.

Figure S13a shows the EELS data for the O-*K* edge and La-$M_{4,5}$ edge for both the interior and surface regions of the nanowire. The spectra were aligned using the O-*K* edge as a calibration. Therefore, the absolute chemical shift of the La-*M* edge can be compared. The chemical shift in EELS is a sensitive fingerprint of the change of oxidation states [S1]. The acquisition of the O-*K* and the La-*M* edges in the same spectrum under the same experimental conditions enables an accurate comparison of the shifts. As the intensity onset of the O-*K* edge is sharp in both the interior and surface spectra, the O-*K* edges can be aligned to single pixel accuracy (0.25 eV). The displacements of the La-*M* edges can then be studied, as shown in Fig. S13c, which is a magnified region (820-870 eV) of the La-*M* edges in Fig. S13c. The peak positions of the La-$M_{4,5}$ edges of the surface spectrum show a shift towards lower energy loss, which indicates a lower oxidation state of La [S2-S4]. The small shift was measured to be ~0.5 eV, suggesting the oxidation state of La is decreased slightly from +3, but is still much larger than +2. It

should be mentioned that the comparison on the absolute peak positions provides a measure of the energy loss of excitations that are closely related to the energy levels of the inner-shell electrons and their final states, and importantly, such transitions in positions are not thickness dependent [S2]. Hence, the relative changes measured here are expected to be a reliable way of discriminating the different chemical states of the interior and surface of the nanowires. Moreover, the change of the oxidation states can be further confirmed using the well-known white-line ratio method [S1,S2,S5], which is undoubtedly the most popular method to relate EELS spectra to the oxidation states of transition metals with sharp $L_{2,3}$ or $M_{4,5}$ edges (the eponymous "white line"). As the white-line ratio is dependent on sample thickness [S2], the effect of thickness must be reduced or removed before calculation. To obtain the correct white-line ratio, the thickness-related multiple inelastic scattering was removed from the EELS spectra by Fourier ratio deconvolution [S1,S2,S6], using low-loss EELS spectra recorded under identical experimental conditions. The background intensity of the spectra was subtracted using a power-law function. Subsequently, determination of the La M_5/M_4 ratio followed the Pearson method [S5]. The remaining background intensity under the La white-lines was subtracted using a two-step baseline function [S2,S5,S6]. Finally, the white-line ratio was determined as the intensity ratio of the M_5 to M_4 peaks, which was obtained by integrating the remaining intensity in a given window width [S2]. The variation in white-line ratio from the surface to the interior is shown in Fig. S13b. Larger values at the surface indicate the lower oxidation state of La [S2-S4,S6], which is in agreement with the conclusion from the chemical shift method. Therefore, we can conclude that the valence of La at the surface decreases slightly, owing to the presence of oxygen vacancies at the surface, as indicated in the following defect equation using Kröger–Vink notation:

$$3(\mathrm{O}^{2-}_{\mathrm{O}^{2-}})^{\times} + 2(\mathrm{La}^{3+}_{\mathrm{La}^{3+}}) \rightarrow \frac{1}{2}\mathrm{O}_2 + (\mathrm{V}_\mathrm{O})^{\cdot\cdot} + 2(\mathrm{La}^{2+}_{\mathrm{La}^{3+}})' + 2(\mathrm{O}^{2-}_{\mathrm{O}^{2-}})^{\times} \qquad (1)$$

Furthermore, as listed in Table S4, the values of average integrated ratio of surface region to interior area, calculated from the La-$M_{4,5}$ and O-K edges, show that the concentration of La atoms on the surface is lower than in the interior of the nanowire, due to a thickness effect. However, the signal strength of O at the surface decreases more rapidly than for

La, which again implies the presence of an oxygen deficiency at the surfaces (in the SAFs).

In summary, the EELS analysis supports the hypothesis that oxygen vacancies or vacancy-like complexes form in the partially ordered region in the glass-like SAFs near the crystalline grains, leading to enhanced ionic conduction.

Supplementary Discussion 3. Discussion of Surface "Amorphous" Films (SAFs)

As shown in Figs. 3c-e in the main text, surface "amorphous" films (SAFs) of a nearly constant thickness of about 3 nm, akin to SAFs observed in other oxides [28-31,S7-S13], were observed to form on the surfaces of LMO nanowires. It is important to note that these SAFs are in fact not fully amorphous, despite their name. Prior studies have shown that such SAFs exhibit an "equilibrium" thickness in the range of 0.5-5 nm in response to a balance between several attractive and repulsive interfacial forces/pressures acting on the films. It is important to note also that the existence of analogous equilibrium-thickness intergranular films (IGFs) was originally proposed by Clarke [S14,S15], which motivated the discovery of SAFs; see a *Critical Review* [S16] and references therein. Cannon *et al.* [S17] proposed that SAFs and IGFs can be alternatively and equivalently explained as a class of disordered multilayer adsorbates with the film thickness and average film composition set by the bulk chemical potentials.

The SAFs and IGFs, along with their discrete derivatives such as the six Dillon-Harmer complexions [S18-S25], are, according to **Defay and Prigogine**'s terminology [32], **"non-autonomous" interfacial phases**, which are thermodynamically two-dimensional (since their effective thickness and through-thickness gradients in structure and composition are thermodynamically determined). More recently, Tang, Carter and Cannon further introduced the terminology of "**complexions**" to describe such "non-autonomous" interfacial phases, based on an argument that they are not "phases" according to Gibbs' rigorous definition [S23,S24]. The concept has been further elaborated in a series of recent publications [28,S23-S39], including two recent comprehensive review articles [33,S25].

Specially, SAFs [28-31,S7-S13] can be considered as a special class of “non-autonomous” surface phases (or complexions). The formation of these amorphous-like surface complexions (SAFs) is driven by a reduction in surface energy (presumably, the crystalline surface can reduce its excess free energy by disordering/amorphization near the surface because a structurally-disordered terminated surface has a lower excess surface energy). Ref. [28] in the main text contains a detailed discussion of the underlying thermodynamic theories for the spontaneous formation of such amorphous-like surface complexions, and specifically nanoscale SAFs. In our studies, XPS, EDXS and EELS revealed no detectable metal impurities in addition to Mo and La (see e.g. Fig. S12), in contrast to observations for many other impurity-based SAFs, although we cannot completely rule out the possible presence of trace impurities below the detection limits of the XPS, EDXS and EELS measurements. However, we wish to emphasize that similar amorphous-like surface complexions (nanoscale SAFs) have been previously observed in impurity-free Al-Mo-O systems; see Refs. [28,S11]. Theoretically, the formation of amorphous-like surface complexions (specifically nanoscale SAFs) is more possible/likely for complex oxides that have two or more cation species (such as $La_2Mo_2O_9$ in the current study, and the Al-Mo-O compound reported previously in Ref. S53), as this favors the interfacial mixing and disordering (see Ref. [28] in the main text for some related theories and discussion of this point).

Recently, SAFs [28-31] and other types of non-autonomous surface phases (complexions) [S30] have been used to enhance the rate capability and cycling stability of lithium-ion battery materials. Moreover, SAFs have also been used to improve the visible-light adsorption and activity of TiO_2 photocatalysts [S31,S32]. In the current case, SAFs serve as a fast ionic conduction pathway, where the conduction in the partially-ordered region in the SAFs near the crystalline grains can be further enhanced by the presence of the strain on the curved surfaces of the nanowires; see Fig. 4f and related discussion in the main text.

In addition to the possibility for enhancement of the rate capability of lithium-ion battery electrodes [28-31,S30], a prior study has also shown that the proton conductivity in solid electrolytes can be enhanced in IGFs [34]. The possibilities of interfacial engineering of

solid electrolytes via the use of non-autonomous interfacial phases (complexions) have been recently reviewed and discussed [39]. Specifically, SAFs (and analogous IGFs at grain boundaries) can have structures that are neither fully amorphous nor completely crystalline [28,33,35-36,S16,S23-S28]. This is because the crystal surface can impose partial order on the nanometer-thick amorphous-like surface complexion (Fig. 3f) [28,33,35-36,S16,S23-S28]. Furthermore, recent theoretical [37] and experimental [38] studies have suggested that ionic conduction can be enhanced along the crystal-glass interfaces in such partially ordered regions, leading to conductivities that can be greater than those of both the crystal and glass phases. Thus, we hypothesize that the observed increased in ionic conductivity of the LMO nanowires is due to enhanced ionic transport in the nanoscale SAFs, particularly in the partially ordered region near the crystalline grains, and moreover that the conductivity can be significantly further enhanced on highly curved surfaces with tensile strains along the surface complexion, as shown in Fig. 3f and discussed in detail in the model presented below.

Supplementary Discussion 4. Analysis of Radius-Dependent Conductivity

We recognize that many possible mechanisms, such as space-charge, strain, and other effects, can lead to enhanced ionic conductivities [39-44]. More recent studies also revealed the effects of change of surface energy and defect presence [26] and carrier-carrier coulombic [45], which were discussed in the main text. In the current case, we further observed curvature-dependent excess conductivities, which could not be explained by prior models; we propose that the curvature-dependent enhanced ionic conductivities can be best explained by the fast conduction in nanoscale, partially-ordered, SAFs strained on curved surfaces (see Fig. 3f in the main text), supported by the HRTEM observation and measured conductivities as a function of the radius. This model is elaborated below.

The enhanced ionic conductivity observed in LMO nanowires (Table S4) is related to the enhanced surface conduction and a size effect. In Fig. S14, we plot the conductivity of the nanowires as a function of the surface-to-volume ratio and the area fraction of the 3-nm-thick SAFs. It is evident from these plots that the enhanced ionic conductivity is not a “trivial size effect” as defined by Maier [40-43], for which the increased conduction

would be linearly proportional to the surface-to-volume ratio (Fig. S14a). The enhanced ionic conductivity increases, in fact, faster with decreasing radius than predicted by a simple composite rule (Fig. S14b). Thus, the observed non-linear increase in ionic conductivity in the LMO nanowires can be classified as “a true size effect” as defined by Maier [40-43]. It should be noted that where enhanced conductivity results from increased carrier concentrations in the space-charge zones, an accelerated increase of conductivity is expected for a Mott-Schottky case, but not for a Gouy-Chapman case; please refer to Ref. S78 for detailed discussion.

Furthermore, in the current observations, the more than one-hundred-fold increase in ionic conductivity cannot be explained by enhanced carrier concentration in the space-charge region, as previously suggested by Maier and co-workers [40-43], because the oxygen vacancy concentration is already high in β-LMO. In the following text we offer an alternative explanation for the origin of the non-linear increase in the ionic conductivity in LMO nanowires.

As shown in the inset of Fig. 4d, the nanowire structure (radius is r) can be regarded as consisting of an interior region (of radius r-δ) and a surface region of thickness δ, where we assume, for simplicity, that conductivity *vs.* radius is represented by a step function. Thus, the total conductivity, σ_{total}, of a LMO nanowire includes two contributions:

$$\sigma_{total} = \sigma_{bulk}\cdot(1 - \delta/r)^2 + \sigma_{surf}\cdot[1 - (1-\delta/r)^2], \quad (2)$$

where σ_{bulk} and $\sigma_{surface}$, respectively, are the bulk and surface conductivities. It is well established that the ionic conductivity is given by:

$$\sigma = nZe\mu, \quad (3)$$

where n is the carrier concentration, Ze is the charge per carrier, and μ is the mobility, which follows an Arrhenius equation of the form:

$$\mu = \mu_0\exp(-E/kT), \quad (4)$$

where E the activation energy (rigorously enthalpy at a constant-pressure condition), k the Boltzmann constant, T is temperature, and μ_0 is pre-exponential effect.

Equations. (3) and (4) can be applied to both bulk and surface (or interface) conduction. Here, we assume that the enhanced surface conduction results from enhanced ionic transport in the partially-ordered region in the SAFs near the crystalline grains (Fig. 3), where partial layering and lateral order exists (Fig. 3f in the main text) [28,33,35-36,S16,S23-S28]. Both theory [37] and experiments [38] suggest that ionic conduction can be greatly enhanced in the partially-ordered region near the crystal-glass interface, and that this can lead to a conductivity that is greater than both the crystal and glass phases. In such a case, we expect:

$$\sigma_{\mathrm{surf}} \propto n_0 \cdot \exp(-E_{\mathrm{m}}/kT) \tag{5}$$

where n_0 is the effective charge carrier concentration and E_{m} is the activation energy for carrier migration along the surface (i.e., in the partially-ordered region in the glass-like SAFs near the crystalline grains; see Fig. 3f in the main text).

In nanowires, conduction along SAFs can be affected by the curvature, thereby resulting in a true size effect. Specifically, the curvature can affect the conduction via two possible mechanisms. First, the curvature may reduce the activation energy for the carrier migration according to:

$$E_{\mathrm{m,\ curv}} = E_{\mathrm{m,\ flat}}(1 - \alpha \cdot \kappa) \tag{6}$$

where $\kappa = 1/r$ is the curvature of the wire surface, and α is a constant. The above equation is supported by a prior theoretical analysis of diffusion on a curved surface [46]. Combining Eqs. (5) and (6) produces:

$$\sigma_{\mathrm{surf}} = \sigma_{\mathrm{surf,\ 0}} \cdot \exp(B/r), \tag{7}$$

where $\sigma_{\mathrm{surf,\ 0}}$ is the conductivity long an SAF on a flat surface and B ($=\alpha/kT$) is a constant. Alternatively, the curvature may also increase the ionic conductivity by increasing the effective concentration of carriers (oxygen vacancies or vacancy complexes in the partially-ordered region in the glass-like SAFs). It is generally believed that the defect (carrier) concentration is related to the defect formation energy (E_{f}) by

$$n_0 \propto \exp(-E_{\mathrm{f}}/kT), \tag{8}$$

and, to a first order approximation, we can again express the effect of curvature as:

$$E_{\mathrm{f,\,curv}} = E_{\mathrm{f,\,flat}}(1 - \alpha^{*} \cdot \kappa). \tag{9}$$

Thus, Eq. (7) can also be used to describe this second possible effect of the curvature on the surface/interface conductivity.

By combination of Eqs. (2) and (7), an expression for the total conductivity at a given temperature can be deduced as:

$$\sigma_{\mathrm{total}} = (1 - \delta/r)^2 \cdot \sigma_{\mathrm{bulk}} + (2\delta/r - \delta^2/r^2) \cdot \sigma_{\mathrm{surf,\,0}} \cdot \exp(B/r). \tag{10}$$

To fit our experimental data collected at 500 °C and 600 °C to Eq. (10), we assume as a simplification that $\delta = 3$ nm (*i.e.*, a constant σ_{surf} through the thickness of the 3-nm-thick SAF), although we expect the conduction should be non-uniform through the thickness of the SAF. At 600 °C, we take the measured bulk conductivity of the β phase as σ_{bulk} (= 6.66×10^{-3} $\mathrm{Scm^{-1}}$); at 500 °C where the bulk specimen transforms to the α phase, we extrapolate the conductivity of the β phase from high-temperature data by assuming an Arrhenius relation, thereby obtaining a value of $\sigma_{\mathrm{bulk}} = 1.23\times10^{-3}$ $\mathrm{Scm^{-1}}$ (whereas the actual measured conductivity for the bulk α phase is 8.38×10^{-5} $\mathrm{Scm^{-1}}$). Then, we rewrite Eq. (10) as:

$$\ln\{[\sigma_{\mathrm{total}}^{(\mathrm{measured})} - (1 - \delta/r)^2 \cdot \sigma_{\mathrm{bulk}}]/(2\delta/r - \delta^2/r^2)\} = \ln(\sigma_{\mathrm{surf,\,0}}) + B/r\ , \tag{11}$$

from which a linear regression analysis can be used to obtain $\sigma_{\mathrm{surf,\,0}}$ and *B*. Specifically, we applied this model to the measured conductivity data at 500 °C and 600 °C. Curves for conductivity *vs.* radius at these two temperatures computed using this model, are shown in Fig. S15 together with the experimental data, where reasonably good agreement is evident. It can be noted for both cases that the surface conductivity for a flat surface is about 12-16 times higher than the bulk conductivity, while the remaining (nonlinear) increase of conductivity is due to the curvature effect.

It can be seen from Eq. (10) that the total electrical conductivity approaches the bulk conductivity (i.e., $\sigma_{\text{total}} \rightarrow \sigma_{\text{bulk}}$) when the radius is very large ($r \rightarrow +\infty$). Moreover, when the radius is sufficiently small, the total electrical conductivity can be written as:

$$\sigma_{\text{total}} \propto \sigma_{\text{surf}} \cdot \exp(B/r). \tag{12}$$

This exponential relationship is evident in Fig. 4d. As shown in Fig. S11, the reduced activation energy for the nanowires compared with the bulk materials soundly supports the above theory.

Finally, we should note that this model, which assumes that the conductivity is uniform through the thickness of the SAFs, is only an approximation for illustration of the key physical idea of the underlying mechanism. Nonetheless, the observed good agreement (Fig. S15) illustrates that the proposed curvature effect can be used to successfully explain the observed non-linear increase of conductivity seen in the LMO nanowires (as a "true size effect", as defined by Maier).

Supplementary References

1. R. F. Egerton. Electron Energy-loss Spectroscopy in the Electron Microscope. Springer (2011).
2. H. Tan, J. Verbeeck, A. Abakumov, G. Van Tendeloo. Oxidation State and Chemical Shift Investigation in Transition Metal Oxides by EELS. *Ultramicroscopy* **116**, 24 (2012).
3. H. Xu, Y. Wang. Electron Energy-loss Spectroscopy Study of Oxidation States of Ce and U in Pyrochlore and Uraninite-natural Analogues for Pu- and U-bearing Waste Forms. *J. Nucl. Mater.* **265**, 117 (1999).
4. J. Bentley, S. R. Gilliss, C. B. Carter, J. F. Al-Sharab, F. Cosandey, I. M. Anderson, P. J. Kotula. Nanoscale EELS Analysis of Oxides: Composition Mapping, Valence Determination and Beam Damage. *J. Phys.: Conf. Ser.* **26**, 69 (2006).
5. D. H. Pearson, C. C. Ahn, B. Fultz. White Lines and *d*-electron Occupancies for the 3*d* and 4*d* Transition Metals. *Phys. Rev. B* **47**, 8471 (1993).
6. D. –M. Kepaptsoglou, K. Hadidi, O. Løvvik, A. Magraso, T. Norby, A. E. Gunnæs, A. Olsen, Q. M. Ramasse. Interfacial Charge Transfer and Chemical Bonding in a Ni-LaNbO4 Cermet for Proton-conducting Solid-oxide Fuel Cell Anodes. *Chem. Mater.* **24**, 4152 (2012).
7. H. J. Qian, J. Luo, Y. M. Chiang, Anisotropic wetting of ZnO by Bi_2O_3 with and without Nanometer-Thick Surficial Amorphous Films. *Acta Mater.* **56**, 862 (Feb, 2008).
8. H. Qian, J. Luo, Nanoscale Surficial Films and a Surface Transition in V_2O_5-TiO_2-Based Ternary Oxide Systems. *Acta Mater. 56*, 4702 (2008).
9. H. J. Qian, J. Luo, Vanadia-Based Equilibrium-Thickness Amorphous Films on Anatase (101) Surfaces. *Appl. Phys. Lett.* **91**, 061909 (2007).
10. J. Luo, Y.-M. Chiang, R. M. Cannon, Nanometer-Thick Surficial Films in Oxides as A Case of Prewetting. *Langmuir* **21**, 7358 (2005).
11. J. Luo, Y. -M. Chiang, Existence and Stability of Nanometer-thick Disordered Films on Oxide Surfaces. *Acta Mater.* **48**, 4501 (2000).
12. J. Luo, Y.-M. Chiang, Equilibrium-thickness Amorphous Films on {11-20} Surfaces of Bi_2O_3-Doped ZnO. *J. Eur. Ceram. Soc.* **19**, 697 (1999).
13. M. Baram, W. D. Kaplan, Intergranular Films at Au-sapphire interfaces. *J. Mater. Sci.* **41**, 7775 (2006).
14. D. R. Clarke, T. M. Shaw, A. P. Philipse, R. G. Horn, Possible Electrical Double-Layer Contribution to the Equilibrium Thickness of Intergranular Glass Films in Polycrystalline Ceramics. *J. Am. Ceram. Soc.* **76**, 1201 (1993).
15. D. R. Clarke, On the Equilibrium Thickness of Intergranular Glass Phases in Ceramic Materials. *J. Am. Ceram. Soc.* **70**, 15 (1987).
16. J. Luo, Stabilization of Nanoscale Quasi-Liquid Interfacial Films in Inorganic Materials: A Review and Critical Assessment. *Critical Reviews in Solid State and Material Sciences* **32**, 67 (2007).
17. R. M. Cannon et al., Adsorption and Wetting Mechanisms at Ceramic Grain Boundaries. *Ceramic Transactions (Grain Boundary Engineering in Ceramics)* **118**, 427 (2000).

18. S. J. Dillon, M. Tang, W. C. Carter, M. P. Harmer, Complexion: A New Concept for Kinetic Engineering in Materials Science. *Acta Mater.* **55**, 6208 (2007).
19. S. J. Dillon, M. P. Harmer, J. Luo, Grain Boundary Complexions in Ceramics and Metals: An Overview. *JOM* **61**, 38 (2009).
20. J. Luo, H. Cheng, K. M. Asl, C. J. Kiely, M. P. Harmer, The Role of a Bilayer Interfacial Phase on Liquid Metal Embrittlement. *Science* **333**, 1730 (2011, 2011).
21. M. P. Harmer, The Phase Behavior of Interfaces. *Science* **332**, 182 (2011).
22. M. P. Harmer, Interfacial Kinetic Engineering: How Far Have We Come Since Kingery's Inaugural Sosman Address? *J. Am. Ceram. Soc.* **93**, 301 (2010).
23. M. Tang, W. C. Carter, R. M. Cannon, Grain Boundary Transitions in Binary Alloys. *Phys. Rev. Lett.* **97**, 075502 (2006).
24. M. Tang, W. C. Carter, R. M. Cannon, Diffuse Interface Model for Structural Transitions of Grain Boundaries. *Physical Review B* **73**, 024102 (2006).
25. W. D. Kaplan, D. Chatain, P. Wynblatt, W. C. Carter, A review of Wetting versus Adsorption, Complexions, and Related Phenomena: the Rosetta Stone of Wetting *J. Mater. Sci.* **48**, 5681 (2013).
26. M. Baram, D. Chatain, W. D. Kaplan, Nanometer-Thick Equilibrium Films: The Interface between Thermodynamics and Atomistics. *Science* **332**, 206 (2011).
27. J. Luo, Grain boundary complexions: The interplay of premelting, prewetting, and multilayer adsorption. *Appl. Phys. Lett.* **95**, 071911 (2009).
28. S. J. Dillon, M. P. Harmer, J. Luo, Grain Boundary Complexions in Ceramics and Metals: An Overview. *JOM* **61 [12]**, 38 (2009).
29. S. Ma *et al.*, Grain boundary complexion transitions in WO_3- and CuO-doped TiO_2 bicrystals. *Acta Mater.* **61**, 1691 (2013).
30. M. Samiee, J. Luo, A Facile Nitridation Method to Improve the Rate Capability of TiO_2 for Lithium-Ion Batteries. *J. Power Sources* **245**, 594 (2014).
31. X. B. Chen, L. Liu, P. Y. Yu, S. S. Mao, Increasing Solar Absorption for Photocatalysis with Black Hydrogenated Titanium Dioxide Nanocrystals. *Science* **331**, 746 (2011).
32. M. Samiee, J. Luo, Enhancing the Visible-Light Photocatalytic Activity of TiO_2 by Heat Treatments in Reducing Environments. *Mater. Lett.* **98**, 205 (2013).
